# Factors influencing the implementation, adoption, use, sustainability and scalability of mLearning for medical and nursing education: a systematic review protocol

**DOI:** 10.1186/s13643-016-0354-x

**Published:** 2016-10-19

**Authors:** Charmaine Krishnasamy, Sik Yin Ong, Yvonne Yock, Issac Lim, Rebecca Rees, Josip Car

**Affiliations:** 1 HOMER, Education Development Office, National Healthcare Group, Tan Tock Seng Hospital, 7 Jalan Tan Tock Seng, Annex 2 (Level 3, West Wing), Singapore, 308440 Singapore; 2 Evidence for Policy and Practice Information and Co-ordinating Centre (EPPI-Centre), Social Science Research Unit, Department of Social Science, UCL Institute of Education, University College London, 18 Woburn Square, London, WC1H0NR UK; 3 Centre for Population Health Sciences, Lee Kong Chian School of Medicine, Nanyang Technological University, Singapore, 3 Fusionopolis Link, #06-13, Nexus@One-North, South Tower, Singapore, 138543 Singapore; 4 Global eHealth Unit, Department of Primary Care and Public Health, Imperial College London, London, UK

**Keywords:** Mobile learning (mLearning), Medical education, Nursing education, Systematic Review

## Abstract

**Background:**

mLearning is increasingly presented as an attractive novel educational strategy for medical and nursing education. Yet, evidence base for its effectiveness or factors which influence use, success, implementation or adoption are not clear. We aim to synthesise findings from qualitative studies to provide insight into the factors (barriers and facilitators) influencing adoption, implementation and use of mobile devices for learning in medical and nursing education. The review also aims to identify factors or actions which are considered to optimise the experience and satisfaction of educators and learners in using mobile technologies for medical and nursing education and to identify strategies for improving mLearning interventions for medical and nursing education.

**Methods:**

A systematic search will be conducted across a range of databases for studies describing or evaluating the experiences, barriers, facilitators and factors pertaining to the use of mLearning for medical and nursing education. The framework synthesis approach will be used to organise and bring different components of the results together. The confidence in the qualitative review findings will be assessed using the CERQual approach.

**Discussion:**

This study will contribute to the planning and design of effective mLearning and the development of mLearning guidelines for medical and nursing education.

**Systematic review registration:**

PROSPERO CRD42016035411

**Electronic supplementary material:**

The online version of this article (doi:10.1186/s13643-016-0354-x) contains supplementary material, which is available to authorized users.

## Background

In the past five decades, eLearning (electronic learning) has increasingly been used in health professional education, and technological advances have produced various forms of eLearning modalities such as computer-based simulations, virtual patients and internet-based courses and interactive content [1]. Adoption of these educational strategies in health professional education is substantial, fast growing and yet appears to be ahead of establishing a robust evidence base for consideration of multiple dimensions and outcomes [2, 3]. A noteworthy trend within eLearning is mLearning (mobile learning) defined as “any activity that allows individuals to be more productive when consuming, interacting with or creating information, mediated through a compact digital portable device that the individual carries on a regular basis, has reliable connectivity and fits in a pocket or purse” [4]. This is enabled by growth of capabilities in mobile devices (e.g. laptops, smartphones, tablets) and the convenience they offer, such as omnipresent usability and accessibility to the internet, whilst mobile. Approximately one third of the 3.4 billion people living in rural areas [5] and 73 % of the total world population [6] are now covered by mobile broadband.

mLearning can provide access to educational content and information in daily clinical practice [7–9]; enable conversations and the sharing of information and knowledge with other learners; and elicit support from peers and instructors regardless of geographic distance [7–9]. Handheld computers can be used to keep track of students’ skill development and progress in assignments [10]; promote self-directed and self-regulated learning [11, 12]; display audio-visual information relating a specific place, scene or situation; and inform situated learning [9].

Evaluations of the effects of eLearning more widely and specifically mLearning as a whole raise more questions than they answer. For example, a meta-analysis by Free and colleagues [13] included seven randomised controlled trials (RCTs) and investigated the educational outcomes associated with the use of personal digital assistants (PDAs) and portable media players in medical and nursing education. These studies looked at the effectiveness of mLearning in improving the knowledge and attitudes; however, the meta-analysis showed no clear evidence of benefit. There are many factors influencing the effectiveness of interventions that warrant closer investigation. For instance, students’ perceived attributes of the intervention, such as attitudes and satisfaction, can contribute to successful implementation [14].

Some factors influencing implementation and adoption of eLearning interventions include characteristics of the educational intervention, problem addressed by the intervention, features of the health system, the adopting system and other contextual factors [14]. A number of qualitative studies have been conducted to evaluate factors affecting implementation and adoption of mLearning interventions. However, no qualitative review has been done to systematically evaluate the factors influencing the successful implementation and adoption of mLearning interventions for medical and nursing education. Our review considers the broader issues of implementation, adoption, and the educational impact of mLearning. This is important because mLearning is a relatively new area of development compared to other eLearning approaches and having a systematic and in-depth exploration of the range of potential barriers and facilitators to adoption, use, scalability and sustainability of mLearning in health professional education would deepen understanding of the topic and allow insights to be obtained. It is also important to understand mLearning in terms of the underlying assumptions about teaching and learning (pedagogy) of different approaches so as to maximise the potential richness of the learning process and enable teachers to plan for optimal learning [15].

Systematic methods can be applied to identify, appraise and synthesise the findings of studies designed to gain insights into people’s experiences of and perspectives on different approaches. Such studies can allow us to better understand the nature of material and socio-cultural influences (e.g. cultural norms), effectiveness of interventions and help us better understand causal pathways [16], or delineate a more complete picture of the phenomenon under study [17]. These studies also take an interpretive approach thus often collect data using flexible methods (e.g. open-ended interviewing and/or observations) and apply qualitative analysis techniques to provide insights into important concepts and to develop theories. The synthesis of such studies tends also to work mainly with qualitative data (from study reports) and configure study findings to produce themes which may be ordered to describe variation within a phenomenon or to be developed into a new theory [18].

An initial scoping of the literature has identified more than 40 studies of factors (barriers and/or facilitators) that are potentially influencing mLearning in health professional education, which examine a range of different mLearning approaches from various countries and cover undergraduate and post graduate programmes. However, no review has been identified, which systematically evaluates the factors influencing the use of mLearning interventions for health professional education.

### Review aims and research questions

The proposed systematic review aims to draw lessons from empirical research that studied the processes involved in the delivery of mLearning in the field of medical and nursing education or has sought the perspectives of learners, educators and others with experience of mLearning in this field. Themes arising from a synthesis of the findings of this research will be used to consider the teaching pedagogies that teachers using mLearning can adopt to optimise knowledge formation and retention, as well as strategies that might ameliorate negative factors and enhance positive factors potentially influencing mLearning for medical and nursing education.

The broad research question for this review is:

What are the views of educators, learners, and other key actors with experience of mLearning in medical and nursing education, about perceived factors which facilitate/enhance or hinder its implementation, adoption, scalability, sustainability and educational impact?

## Methods/design

Given that the extent of the evidence base that addresses the above research question is unknown; this review will have two stages: a systematic ‘mapping’ of research evidence, followed by an in-depth analysis and synthesis. An initial mapping stage, which tends to use wide-ranging searches and broad inclusion criteria, can be used to help review teams identify a wide range of types of study that can then be screened systematically again, once more is known about the nature of the existing evidence base [19]. In the case of this review, the broad inclusion criteria for the map will enable identification of studies regardless of whether they are labelled by their authors as ‘qualitative’. It may then be appropriate to synthesise all of the studies identified by the map or to select a smaller number that meets a tighter set of inclusion criteria, for example, a requirement that studies use both qualitative data collection and qualitative data analysis methods.

Amongst the many conceptual frameworks that could be validly used for synthesis in this review, we considered Laurillard’s comprehensive Conversational Pedagogical Framework. Laurillard’s framework describes interactions between learners, peers and teachers in both formal and informal learning contexts [15]. It provides a detailed description of components affecting the motivation of the learners in collaborative learning environments using a combination of social learning theories, constructionism and instructionism. The model sees each teacher, each student and the other students they work with as being influenced by each other’s presentations of concepts and responses to learning tasks and goals and also attempts to capture ideas about the influence of teachers’ designs for learning environments as well as learners’ and peers’ responses to each other’s practices within these environments. This model, however, does not consider the influence that socio-cultural constructs can play on the mLearning process in the medical education context. It has been advocated that medical education researchers and curriculum developers consider the external, internal, implementation, experience and impact factors when designing technology-based interventions [20].

Another conceptual model that might inform this review’s analyses is the one developed by Tabak and Nguyen [21]. This integrates three theories from different domains: the technology acceptance model (TAM) [22], the concept of self-regulation from social cognitive theory [23] and the five-factor model of personality [24] to integrate both intrinsic and extrinsic influences on learners within online learning environments. This middle-range theory might help with exploration of the types of situational variables influencing mLearning processes at the individual learner level.

### Criteria for considering studies for this review

Inclusion and exclusion criteria will be required for two different purposes. Firstly, the search results will be screened to identify studies to include in an initial systematic map. These studies will be coded (see below) so as to provide an overview of the nature and extent of the literature that addresses the review’s research questions. Following the consideration of the range of study designs seen in the map, the mapped studies may be screened again using a refined set of inclusion criteria so as to identify studies that have applied certain aspects of study design, with only these studies then being described further, being fully appraised for their methodological quality and having their findings extracted for inclusion in a synthesis.

#### Types of studies

To be included in the systematic map, study reports will need to:

- Have an abstract in English
- Be conducted in any geographical setting (e.g. low, middle and high-income countries), involving all types of healthcare (e.g. primary, secondary, tertiary) and educational setting (e.g. university, laboratory, medical ward, community)
- Be a report that presents findings or analyses of data from a primary research study (Systematic reviews that meet all other criteria will not be used as a source of data but their reference lists will be screened for other suitable studies. Commentaries, letters, editorials and other kinds of literature reviews will be excluded).
- Be a study that examines people’s perspectives on and experiences of mLearning (see intervention below) so as to produce findings about perceived factors which facilitate/enhance or hinder its implementation, adoption, scalability, sustainability and educational impact. For in-depth review and synthesis, depending on the quantity and nature of the research found in the systematic map, it may be helpful to restrict studies to those that meet the inclusion criteria above and below but also:
- Collect and analyse data primarily through the use of qualitative methods.
  - ○ This would include studies underpinned by theoretical frameworks such as phenomenology, ethnography and grounded theory, as well as many forms of action and narrative research and case studies. Qualitative methods for data collection would include focus groups, in-depth individual interviews and observations.

#### Types of participants

- The studies should include participants who are or have been enrolled in a pre- or post-professional, undergraduate or postgraduate, medical or nursing professional education programme.

#### Types of interventions

The studies should explore mLearning interventions in pre- and post-qualification medical or nursing education or mLearning being implemented in educational settings for medical and nursing professionals:

- ○ Where mLearning is defined as earlier above. This includes interventions which use mobile phones, PDAs, PDA phones, smartphones, pocket computers, handheld and ultra-portable computers such as tablet personal computers (PCs)
- ○ Where it is clear that reference is being made specifically to mLearning (for example, not solely to a mixture of mLearning and non-mLearning interventions, in which both types of learning components have been received)

#### Search methods for identification of studies

This study is part of a larger series of evidence synthesis reviews on eLearning for health professional education conducted in collaboration with the World Health Organization, for which a common search strategy has been developed. Hence, the searches for this review will be run as one component of a larger group of related reviews that also examine the effects of eLearning interventions and barriers and facilitators of eLearning of all types within education for health professionals. The searches covered the following bibliographic databases:

#### Electronic searches

• Systematic review registers:
  - ○ The Cochrane Library (Cochrane Database of Systematic Reviews, Cochrane Central Register of Controlled Trials (CENTRAL), Cochrane Methodology Register)
  - ○ Joanna Briggs Institute Database of Systematic Reviews and Implementation Reports
• Education focused databases:
  - ○ Education Resources Information Centre (ERIC)
• Health focused databases
  - ○ Cumulative Index to Nursing and Allied Health Literature (CINAHL)
  - ○ EMBASE (Elsevier)
  - ○ MEDLINE (Ovid)
• Other databases
  - ○ PsycINFO
  - ○ Web of Science Core Collection (Thomson Reuters)

When searching these databases, sets of terms were identified from each database’s controlled classifying terminology for each of the main concepts found within all of the reviews’ research questions (eLearning, education, medical and nursing students and professionals). These sets of terms will then be combined to find only those records that have been classified with a term relating to all of the concepts. Searches using free-text terms will also be run to help identify relevant studies that, for whatever reason, have not been allocated controlled terms. Searches will be limited to items published from 1990 onwards. At the time of submitting this protocol, more than 40 studies had been identified using the methods for the systematic map. The studies are described in Additional file 1: Appendix 1.

#### Data collection and analysis

All records of studies that are identified by these searches will be uploaded to the specialist systematic review software EPPI-Reviewer 4 [25], where duplicate studies will be removed, and the remaining studies will be screened. Separate searches for the purposes of this review will then be conducted within EPPI-Reviewer to identify relevant studies. Further sets of terms will be combined, using the software’s search function, which looks for each search term within a record’s title and/or its abstract. Sets of terms will be developed to cover the concepts central to this review (mLearning, perspectives and experiences) and combined, as described above, so as to identify a set of records to screen. The references of included studies will be screened.

#### Selection of studies

All review authors will initially work together with a sample set of identified studies. These will be used to pilot the inclusion criteria and then to reach a high level of concordance between all review authors in using the criteria to determine a study’s eligibility for inclusion. The titles and abstracts of each report retrieved from the search strategy and the additional sources described will then be screened independently by two review authors, who will discuss all cases where they initially disagree on whether or not a report should be included. Full reports will be obtained for those studies that appear at this point to meet the inclusion criteria for the systematic map. Retrieved reports will then be screened again, also by two review authors, working independently. A third review author will help decide upon inclusion of a report in all cases where the two initial reviewers cannot agree.

#### Data extraction and management

All studies that are included in the systematic map will be described according to a standardised coding system modified from one used in previous similar reviews (e.g. Shepherd and colleagues [26]). Codes applied to capture the key characteristics of relevant studies are likely to include but not be limited to:

• Codes to describe the study context and population, including
  - ○ The country setting (e.g. name of country, which will then be classified using the World Bank classification of low-, lower middle-, upper-middle or high-income country)
  - ○ The educational setting (e.g. university/educational institution, healthcare institution/healthcare setting, community/home)
  - ○ Relevant defining features of the sampled population (e.g. gender, age, years of education/training, type/level of training, years of experience, professional group)
• Codes to describe the intervention under study, including
  - ○ Study aims/research questions
  - ○ Learning expectations
  - ○ The mLearning modality (e.g. iPad, iPod, mobile phone, smart phone, tablet, personal computer, PDAs)
  - ○ Learning platform (e.g. chatgroup, e-book, web-based module, mobile application, computer program)
  - ○ Component of intervention (e.g. skills training, education/information/theory, professional training, provision of resources/supplementary information, services)
  - ○ Duration of intervention (e.g. <1 month, 1–6 months, 7–12 months, >12 months)
• Codes to describe the study design, including:
  - ○ The type of data collection method used (e.g. survey with open-ended questions; observational study using case study techniques; in-depth individual interviews; focus groups)
  - ○ Sampling approach (e.g. convenience sampling, random sampling, purposive sampling, snowball sampling, theoretical sampling)
  - ○ The sampling frame (e.g. course enrolment list, directory of doctors/ nurses working in the hospital)
  - ○ The sample size (e.g. <10, 11–20, 21–50, 51–100, >100)
  - ○ The type of analysis (qualitative only or qualitative and quantitative).
  - ○ Type of outcomes (e.g. attitudes, skills, knowledge, experiences, feelings)

Where possible, the mLearning experiences of the students will be compared based on their age, type/level of educational training, professional group, years of education/ training and learning expectations. All studies that are included in the in-depth review will be described further using additional, standard questions, such as those used in previous reviews of intervention processes and stakeholder perspectives (e.g. Rees et al. [27]; Shepherd et al. [26]).

#### Appraisal of study quality and certainty of review findings

The quality of studies included in the in-depth review will be examined using a quality-assessment tool for qualitative studies (a modified version of a Critical Appraisal Skills Program tool [28]. Studies that meet the inclusion criteria will be included in the review regardless of the study quality, and the quality assessment will be used to describe the findings of the study. Each relevant review finding will be assessed using the GRADE-CERQual (Confidence in the Evidence from Reviews of Qualitative Research) approach. The purpose of using the CERQual approach is to assess and describe how much confidence can be placed in findings from systematic reviews of qualitative evidence [29, 30]. The term “confidence” refers to the assessment of the extent to which a finding from the review adequately represents the phenomenon of interest, such that the phenomenon of interest does not differ considerably from the research finding. The CERQual approach is still being refined; hence, the version by Lewin et al. [30] that we will be using will guide assessment of four aspects, namely the methodological limitations of the qualitative studies that contribute to a review finding, the relevance of the studies that inform a review finding, the coherence of the review finding and the adequacy of data supporting a review finding.

#### Data synthesis

The framework analysis approach which is recommended in the literature as a highly structured approach to organising and analysing data will be used to analyse the contextual details and findings from each study. This involves the construction of thematic categories from the findings of included studies through the use of a matrix within which the findings are coded [31]. A distinctive feature of this approach is that an a priori ‘framework’ is used as a starting point for the synthesis. An initial, ‘good enough’, framework is developed from the review team’s reading and discussion of theoretical material that relates to the concepts in the review’s research question. The synthesis approach is then deductive [32], with reviewers attempting to match the findings of included studies with the different aspects of their initial conceptual frame. When the findings are found to address an area not covered in the initial frame, the frame is modified, until the frame addresses all of the themes arising from the included studies. Additional work will be performed to ensure that the synthesis takes into account the variation across and within different study populations and contexts. The following five stages of framework analysis identified by Pope, Ziebland and Mays [33] will be applied:

1. Familiarisation—this stage involves the authors being immersed in the data by reading and studying the papers retrieved with the aims and objectives of the review and listing key ideas and recurrent themes.
2. Identifying a thematic framework—this process involves the identification of key issues, concepts and themes using the a priori issues in the aims, objectives, experiences and perspectives that recur in the data. This stage results in the formulation of a detailed index of the data, in which data is labelled in manageable chunks for subsequent retrieval and exploration. At this stage, we may incorporate into a framework, aspects of Laurillard’s framework [34] so as to identify the different pedagogic forms of eLearning that have previously been described by teachers as optimising learning and aspects of the meso-level framework developed by Tabak and Nguyen [21] to allow consideration of a wider range of intrinsic and extrinsic factors influencing the eLearning process.
3. Indexing—in this stage, the thematic framework is applied by annotating transcripts of findings from included studies with codes from the index and supporting them with short text descriptors to substantiate the index heading. Review authors will independently read and re-read the selected studies and apply the review’s initial framework. The framework can be applied by moving between the data and themes covered by the framework and searching for additional themes until all of the studies have been reviewed, in an iterative manner. At this stage, the definitions and boundaries of each of the emerging themes will be discussed amongst the review authors and revision of the model will be conducted in line with the ideas and categories that emerge from this process.
4. Charting—the data is then re-arranged according to the relevant part of the thematic framework, and the information is distilled and summarised into charts. The charts will contain distilled summaries of evidence from different perspectives and involve a high level of abstraction and synthesis.
5. Mapping and interpretation—finally the charts will be used together with the research objectives and themes that have emerged, to define concepts and explain the findings through clarification of the phenomena, creation of typologies and finding associations between themes.

#### Reporting methods

This protocol is reported according to the Preferred Reporting Items for Systematic Reviews and Meta-analyses (PRISMA)-P Statement for reporting systematic review protocols [35]. A completed PRISMA-P is attached in Additional file 2.

## Discussion

An understanding of factors influencing the implementation, adoption, scalability, sustainability and educational attainment via mLearning is key to maximising its potential and ensure the most appropriate use. In addition, we will identify gaps in literature to inform future research and policy development in this promising area of learning innovation.

## Additional files

Additional file 1: Initial review of articles retrieved from database [36–75]. (DOCX 53 kb)
Additional file 2: PRISMA-P (Preferred Reporting Items for Systematic review and Meta-Analysis Protocols) 2015 checklist: recommended items to address in a systematic review protocol. (DOC 82 kb)
