# Supplementary material for: Factors influencing the implementation, adoption, use, sustainability and scalability of mLearning for medical and nursing education: a systematic review protocol
Source: Syst Rev. 2016 Oct 19;5:178. doi: 10.1186/s13643-016-0354-x (PMC5069977; doi:10.1186/s13643-016-0354-x)
Supplement: Additional file 1: — Initial review of articles retrieved from database [36–75]. (DOCX 53 kb) [file 13643_2016_354_MOESM1_ESM.docx]

# Appendix 1: Initial mapping of articles retrieved from database

| Ref | Author (Year) | Country | Study Design | Research Question/ Aim | Type of device |
| --- | --- | --- | --- | --- | --- |
| [36] | Abate (2013) | USA | Descriptive: Survey | To evaluate the effectiveness of academic podcasts in promoting knowledge retention and application in nursing students. | Computer or MP3 player |
| [37] | Al-Fahad (2009) | Saudi Arabia | Descriptive: Survey | To explore student attitudes and perceptions on the effectiveness of mLearning; to draw the student's preferences for mLearning in distance education; to examine the extent of use of mLearning by distance learners. | Smart phones |
| [7] | Alegria et al (2014) | USA | Descriptive: Focus groups | To understand how students use tablet computers on clinical rotations, and describe how students in a longitudinal integrated clerkship employ tablet computers for self-regulated learning. | Tablet (iPad 2) |
| [38] | Alipour et al  (2012) | Iran | Pre test-post test | To evaluate the impact of using the short message service (SMS) via cell phone in improving the knowledge about breast cancer in residents of Obstetrics and Gynaecology in a hospital and compare it with a paper-based method. | Non smartphone  (mobile phone) |
| [39] | Arvey et al (2012) | USA | Descriptive: Ethnography, in-depth interviews | To fill gaps in knowledge about community health workers for Spanish speakers and their use of small media to increase cancer screening by describing how they adapt to and use different small media formats to increase colorectal cancer screening among medically underserved Latonos; to compare community health workers for Spanish speakers' use of two different types of multimedia tools (low-tech and high-tech) to deliver a colorectal cancer screening intervention to medically underserved Latinos. | DVD player and Tablet computer |
| [40] | Bidaki et al (2013) | Iran | Descriptive: Survey | To evaluate the role of m-books in changing students' attitudes towards mLearning; to evaluate the role of m-books in encouraging students to extend their time of study to dead times and in motion when paper books are less applicable. | Smartphones |
| [41] | Bogossian et al  (2009) | Australia | Descriptive: Focus groups and surveys | To determine the effectiveness of using a digitalised clinical portfolio in an undergraduate nursing program. | Tablet (Hewlett-Packard Compac TC4400) |
| [42] | Boruff & Storie (2014) | Canada | Descriptive: Electronic survey | What is the extent to which medical trainees and faculty use their mobile devices when answering clinical questions and finding medical information; what are the facilitators and barriers to using mobile devices to find information related to medical studies and clinical work; how do health libraries support mobile users' clinical information needs. | Tablet, PDA and Smartphone |
| [43] | Bruce-Low et al (2013) | UK | RCT (Pre & Post-test) & focus group | To examine whether learning using a mLearning device (Samsung NC10 Netbook) loaded with interactive exercises promoted learning compared with a traditional library exercise. | Netbook (Samsung NC10) |
| [44] | Burke and Cody (2014) | USA | Descriptive: Survey | To examine undergraduate nursing students’ perceptions of podcasted materials, determine the benefits of podcasting, and determine when and where students used podcasted materials. | Podcast accessed from home, car, etc) |
| [45] | Chang et al (2012) | Botswana | Descriptive: Survey | To obtain feedback on a smartphone-based mLearning tool which was implemented to help residents train and care for patients by providing access to medical resources and remote mentoring. | Smartphone (myTouch 3G smartphone) |
| [46] | Chib  (2010) | Indonesia | Descriptive: Quantitative survey, in-depth interviews and focus groups | Examine the benefits of mobile usage in the rural healthcare context; specifically opportunity production, capabilities enhancement, social enabling and knowledge generation; examine the inter-related constraints to mobile usage in the rural healthcare context; specifically infrastructural, economic, technological, and socio-cultural factors from an organization perspective. | Non smartphone |
| [47] | Clay  (2011) | UK | Descriptive: Survey and focus group | To identify if the support of a handheld mobile device such as an iPod loaded with the relevant teaching material and reusable learning objects enhanced the mobility of learning for the learner. | PDA  (iPod touch) |
| [48] | Davern et al (2014) | USA | Quizzes and feedback questionnaire | To identify whether mobile-learning can be a solution to practice learning in the face of time and resource constraints | PDA & Tablet (iPod, iPad, and iPod Touch devices) |
| [49] | Davies (2014) | UK | Descriptive: Questionnaire | A mobile technology device (iPad) was introduced in order to increase interactivity and group cohesion within the tutorial/seminar environment. | Tablet  (iPad) |
| [11] | Davies et al (2012) | UK | Descriptive: Surveys, focus groups | How medical students use technology, how it enables them to learn and what theoretical underpinning supports the learning | PDA(Hewlett-Packard iPAQ 114 Classic) |
| [50] | Dearnley et al  (2008) | UK | Descriptive: Focus groups | To explore the feasibility and identify the issues of using mobile technologies in the assessment of health and social care students in practice settings. To identify the readiness of Assessment and Learning in Practice Settings partner institutions to adopt mobile technologies for assessment in clinical settings; identify the infrastructure available and required for support in using mobile technologies; explore the impact of using mobile technologies on current assessment processes and outcomes. | PDA  (HP iPAQ 6500) |
| [51] | Farrell et al  (2008) | Australia | Quasi-experimental and focus group discussion | To ascertain how nursing students used PDAs to enhance contextual learning and the benefits and difficulties associated with their use | PDA  (HP iPAQ Pocket PC h5500) |
| [52] | Gibbons (2009) | UK | Descriptive: Phenomenological case‐study | To give immediate access to mobile visual technologies for a number of specifically targeted students and explore the presumed benefits of visual learning in relation to researcher activity, for these and other postgraduate groups and how having this device changed their learning? | Smartphone  (HTC TyTN Pocket PC) |
| [53] | Han et al (2014) | USA | Descriptive: Survey | To investigate medical students’ technology use, online behaviours, and perceived usefulness of technology for their learning. | Various mobile devices  (including laptops, smartphones) |
| [9] | Hardyman et al  (2013) | UK | Descriptive: Survey and qualitative case report | To assess the value and impact of the smartphone library of texts to support workplace learning. Used Ellaway's framework of factors to explore contextual mLearning and David et al's approach to understand what works, how it works and when it works. | Smartphone |
| [54] | Kazlauskas and Robinson (2012) | Australia | Descriptive: Contingency table analyses & Action Research Methodology | To investigate the anticipated benefit of lecture podcasts to a wide range of students | Various mobile devices for podcast |
| [55] | Kenny (2012) | Canada | Descriptive: Cross-sectional survey | In what ways are faculty and students currently using personal mobile devices in their teaching and learning; how do they foresee using personal mobile devices in teaching and learning in the future; to what degree is the level of mobile self-efficacy of nursing faculty and students related to their potential use of m-technology in teaching and learning | Various mobile devices  (including smartphones, non-smartphones) |
| [56] | Kenny et al (2009) | Canada | Descriptive: Survey and semi-structured interview | To evaluate whether the implementation of mobile devices in an independent nursing practice education setting would be feasible and practical, and to assess if nursing students and instructors would find the use of such devices in this context to be comfortable and helpful in assisting their learning | Smartphone  (HP iPAQ Model 6955) |
| [57] | Kumar (2011) | India | Descriptive: Questionnaire and phone interview | What is the experience of learners towards the use of mobile devices for learner support services; what is the opinion of learners regarding the use of mobile devices in enhancing learning; what is the experience and perception of Programme-in-charges regarding the use of mobile phones for learner support activities and in the enhancement of learning respectively | Non-smartphone |
| [58] | Maag  (2006) | USA | Cross-sectional survey | To present an emerging mLearning tool, podcasting, used in a traditional didactic medical-surgical nursing course and students’ satisfaction with the technology | Various mobile devices for podcast |
| [59] | Martyn et al  (2014) | Australia | Descriptive: Survey, focus group, interview, online discussion forum, reflective journal | How does the introduction of the iPod Touch affect distance learners' interactions with course content; how does the introduction of the iPod Touch affect distance learners' patterns of communication with instructors and peers; which capabilities of the mobile devices are valued by distance learners and for what purposes; what constraints are evident in the use of mobile devices to support distance learning; what modifications to university study resources are necessary or desirable to optimise their use on the iPod Touch or similar mobile devices | PDA  (iPod Touch) |
| [60] | Mather & Cummings (2014) | Australia | Cross-sectional online survey | To examine the preparedness of a group of nurses to develop and engage with a CoP to support their supervision of student nurses during their work integrated practice in a range of healthcare environments | Perceptions of using various mobile learning devices |
| [61] | Mather et al (2014) | Australia | Cross-sectional online survey | To identify differences in behaviour of undergraduate nurses in accessing information, using a portable or mobile device, when undertaking work-integrated learning (WIL) compared to other non-work situations | Various mobile devices |
| [62] | McAlearney et al  (2004) | USA | Descriptive: Focus groups | How and why doctors use hand-held computers in clinical practice; what barriers doctors perceive with their use and how these could be overcome; what doctors expect from their use in the future | Handheld computers |
| [63] | McKinney et al  (2009) | UK | Descriptive: Questionnaire | To report the outcomes of one pilot project which evaluated nursing students views on the inclusion of a variety of multimedia resources alongside more traditional teaching approaches to facilitate the learning of pathophysiology | iPod/ MP3 player for podcast/vodcast |
| [64] | Meade (2009) | UK | Cross-sectional survey | To provide an evaluation of the usefulness of podcasts of pharmacology lectures as a supplementary learning tool for non-medical processing students. Specifically, the focus of this evaluation is on student use of the podcasts on offer, their perceptions of the usefulness of these podcasts and any potential impact on students' pharmacological knowledge. | Various devices or MP3 player for podcast |
| [65] | Nestel (2010) | Australia | Descriptive: Questionnaire & individual interview | Explore students' experiences of mLearning: In what ways does mLearning support learning; what areas need development | Laptop  (HP 610p) |
| [66] | Pilcher et al (2011) | USA | Descriptive: In-depth interviews and questionnaire | To what extent are nurses willing to use technology for ongoing learning; how do nurses' learning preferences vary based on the educational delivery mode; what influence does the type of ongoing education have on nurses' preferences to learn with technology; what role do demographic factors have on nurses' willingness to learn with technology | iPods, Smartphone (BlackBerrys or iPhones) for educational purposes |
| [67] | Pimmer (2014) | South Africa | Descriptive: Semi-structured interviews | Do nurses in disadvantaged and remote areas use mobile phones as effective educational tools, and if so, how and why? | Smart phones |
| [68] | Pimmer (2013) | Nepal | Descriptive: Focus groups | To what extent does the adoption of tools (in the form of new information and communication technology) lead to new and adapted learning activities of undergraduate students and residents in resource constrained environments; to what extent does the adoption of tools (in the form of new ICT) lead to contradictions and changes of rules, communities and division of labour in the respective activity systems? | Smart phones and small laptops |
| [69] | Schrader et al  (2006) | Germany | Descriptive: Questionnaire | To study the impact of the case studies on the learning experience, and their impacts in professional reasoning, professional performance, sustainability and complexity. | Laptop |
| [70] | Strickland (2012) | UK | Descriptive: Electronic questionnaire | Evaluation of the introduction of podcasts in an undergraduate research module in the School of Nursing, Midwifery and Social Care at Edinburgh Napier University | Various devices for podcasts in WebCT |
| [12] | Tanaka et al (2012) | USA | Pre-post evaluation | To compare scores on house staff evaluations of “overall teaching quality” during a rotation in anaesthesia for orthopaedics in the six months before (n = 11 residents were provided curriculum in a printed binder) and in the six months after (n = 9 residents) receiving the same curriculum content in a tablet computer | Tablet (iPad) |
| [71] | Tews et al  (2011) | USA | RCT and post study survey | To evaluate the effect of just-in-time mLearning videos on medical student case presentations in our emergency department. | PDA  (iPod Touch) |
| [72] | Wallace et al (2012) | Canada | Descriptive: Semi-structured interviews and online survey | To examine how medical teachers and learners are using mobile computing devices such as the iPhone in medical education and practice, and how they envision them being used in the future. | PDA and smartphone  (iPhone, Blackberry, Android, HTC, Samsung, Palm, Symbian) |
| [73] | Wang et al (2012) | UK | Descriptive: Semi-structured interviews | To find out the perceptions and uses of a mobile device by a group of mature part-time learners. | Smartphone  (HTC TyTN Mobile Pocket PC) |
| [74] | Wu  (2012) | Taiwan | RCT and questionnaires | To adopt cognitive apprenticeship teaching as a framework to train the students in learning physical assessment standard operating processes via a mastery mechanism in a context-aware mLearning environment for mastering the procedures as experts | Radio-frequency identification (RFID) reader on mobile device |
| [75] | Zolfo  (2010) | Peru | Descriptive: Survey and focus group | To enable healthcare workers involved in HIV/AIDS care in urban and peri urban stations in Peru to access the state-of-the-art in HIV treatment and care | Smartphones |
